# Association between dietary inflammatory index and mental disorders using multilevel modeling with GLIMMIX

**DOI:** 10.3389/fnut.2024.1288793

**Published:** 2024-01-12

**Authors:** Reza Beiranvand, Mohammad Ali Mansournia, Farhad Vahid, Ali-Akbar Nejatisafa, Saharnaz Nedjat

**Affiliations:** ^1^Department of Epidemiology and Biostatistics, School of Public Health, Tehran University of Medical Sciences, Tehran, Iran; ^2^Department of Epidemiology and Biostatistics, School of Public Health, Tehran University of Medical Sciences, Tehran, Iran; ^3^Nutrition and Health Group, School of Heath, Arak University of Medical Science, Arak, Iran; ^4^Psychosomatic Research Center, Department of Psychiatry, Tehran University of Medical Sciences, Iranian Association of Psychosomatic Medicine, Tehran, Iran; ^5^School of Public Health, Tehran University of Medical Sciences, Tehran, Iran

**Keywords:** dietary inflammatory index, mental disorders, multilevel model, GLIMMIX, DiI

## Abstract

**Introduction:**

The Dietary Inflammatory Index (DII) is a composite nutritional index that has gained significant attention in the past decade due to its association with physical and mental well-being. To accurately assess the precise effects of DII on health outcomes, the effects of nutrients and foods need to be adjusted. This study aimed to investigate the association between DII and mental disorders (depression, anxiety, and stress) using multilevel modeling to minimize the bias of the previous methods.

**Methods:**

This cross-sectional analytical study was conducted using data from the initial phase of the Tehran University of Medical Sciences Employees’ Cohort Study (TEC). Nutritional information was obtained through a dish-based semi-quantitative food frequency questionnaire (DFQ), while psychological data were collected using the depression, anxiety and stress scale (DASS-42). The acquired data were analyzed using multilevel modeling in three levels (foods, nutrients, and DII, respectively) through GLIMMIX in the SAS software.

**Results:**

A total of 3,501 individuals participated in this study. The results of the multilevel model demonstrated a significant statistical association between DII and mental disorders after adjusting for baseline characteristics, nutrients and foods. For each unit increase in DII, the mean scores for stress, anxiety, and depression increased by 3.55, 4.26, and 3.02, respectively (*p* < 0.001).

**Conclusion:**

Based on the multilevel model’s findings, it is recommended to minimize the use of pro-inflammatory nutrients and foods to increase the mental health. Multilevel data analysis has also been recommended in nutritional studies involving nested data to obtain more accurate and plausible estimates.

## Introduction

Psychological and emotional disorders play a significant role in the burden of disease, disability, and mortality worldwide, not only due to economic factors but also in association with other chronic physical illnesses (1, 2). According to the latest estimate of World Health Organization (WHO), one in every eight people in the world live with a mental disorder (3). Projections indicate that depression will be the second-leading cause of disease burden globally by 2030 (4, 5). Overall, more than 50% of the general population in countries with average to high incomes will experience at least one mental disorder during their lifetime, indicating that mental illnesses are not limited to a small susceptible group but represent a major public health issue with significant consequences not only for affected individuals but also for their families, social, and work environments (6). In 2015, the prevalence of mental disorders in Iran is estimated at over 23% (7). In 2021, the prevalence of depression, anxiety, and stress among Tehran University of Medical Sciences (TUMS) employees was reported as 30, 26.9, and 41.9%, respectively (8). One of the determinants of physical and mental health is dietary intake/patterns and habits. Recently, nutrition’s modulatory and preventive roles in the inflammatory pathways of mental disorders, including depression, stress, and anxiety, have received particular attention (9, 10).

Given that dietary patterns and habits are complex and subject to change, and most previous studies investigating the association between diet and mental disorders have focused on individual nutrients without considering other nutrients, it seems that these studies have been unable to determine the role of each of these factors alongside the modulatory or interaction effects of other nutrients. Therefore, special attention should be paid to the combined effect of nutrients and foods on mental health (11–14).

One composite nutritional index related to mental disorders is the Dietary Inflammatory Index (DII). DII is a combination of various nutrients and foods, and as a whole, this composite can be related to inflammation and, ultimately, an increased risk of mental disorders. Diets high in sugar, saturated fat, dairy products, and fried foods are associated with increased systemic inflammatory markers such as C-reactive protein and interleukins 1β, 4, 6, and 10, as well as TNF-α (15–20).

However, various studies have shown that the use of conventional analytical methods to analyze the potential association between nutrients, foods, and DII and mental disorders is challenging due to the nested association between them (nutrients within foods and DII within nutrients), high correlations, and a large number of these components. This can lead to potential data outliers or collinearity issues between these factors (21–23). The relationship between DII and mental disorders has been the subject of disagreement and contradiction in various studies (14, 15, 24–31). Since anti-inflammatory and pro-inflammatory foods are consumed together, investigating the relationship between DII and mental disorders presents a challenge (17).

Based on the fact that foods contain nutrients that are not included in the DII and may potentially affect mental disorders, as well as the possibility that the nutrients included in the DII may have properties that impact mental disorders through pathways, other than inflammatory ones, it is necessary to include these foods and nutrients as independent variables in the regression model to control for their potentially confounding effects (32). However, since nutrients are nested within foods, and DII is nested within both nutrients and foods, and there is a high correlation among them, it is essential to use multilevel linear regression models to mitigate the potential confounding and collinearity effects of nutrients and foods in the association between DII and mental disorders, as well as to prevent the occurrence of outliers in order to obtain more accurate and reliable estimates (33). This approach differs from other nutritional studies that evaluate the DII separately and may not be able to capture complex relationships between this index and other dietary components (34).

One way to investigate the relationship between DII and mental disorders is through the use of multilevel regression models. These models can control the confounding and collinearity effects of nutrients and foods, while also preventing the occurrence of outlier data. Therefore, this study aimed to investigate the association between the DII and mental disorders using a multilevel regression model through GLIMMIX. This approach aimed to address the limitations of previous studies and provide more accurate and reliable estimates by investigating the relationship between DII and mental disorders while minimizing confounding and small-sample biases.

## Methods

### Participants and study design

This cross-sectional analytical study used a subset of data from the first phase of the Tehran University of Medical Sciences Employees’ Cohort Study (TEC). In the first phase of the TEC study, 3,550 individuals were examined. This study utilized information from individuals who met the inclusion and exclusion criteria for participation in the study (35). The TEC study has been ethically approved by the ethics committee of Tehran University of Medical Sciences with code numbers IR.TUMS.VCR.REC.1396.4265 and IR.TUMS.VCR.REC.1398.246.

### Eligibility criteria

Participants with any employment status at Tehran University of Medical Sciences and its affiliated centers who consented to participate in the TEC study were included. Individuals who reported ±3 standard deviation kilocalories of daily energy intake were excluded from the study.

### Outcome measurement

In this study outcome variable was mental disorders (depression, anxiety and stress). In the TEC study, the depression, anxiety and stress scale (DASS-42) that consisting of 42 items, was used to measure mental disorders. The answers to the questions were designed in the form of 4 options, which are: never, sometimes, a little and always; and scores were given from 0 to 3, respectively. Finally, each person’s score in all three scales was calculated separately. Based on the scores obtained in each scale, higher scores indicated mental disorders (35, 36).

### Exposure measurement

The main exposure in this study was DII, which was calculated using the nutritional information collected in the TEC study. The TEC study assessed individuals’ nutritional status using a dish-based semi-quantitative food frequency questionnaire (DFQ) comprising 116 food-items. This questionnaire measured the portion sizes and frequency of each food item an individual consumed over the past year. After obtaining nutritional information, the consumption amount of each item was entered into the Nutrition 4 software (N^−4^) to calculate the nutrient intake per 100 grams of consumed food (37).

Next, the DII, which comprises inflammatory-related dietary parameters, was calculated (16). It is worth mentioning that the DII is a flexible index, and depending on the available data, a specific number of parameters can be used to calculate it in different studies. According to validation reports, using even only 21 out of 45 items can correctly predict serum inflammatory biomarkers (38). In this study, the design and method of collecting nutritional information (instead of using the 24-Hour Food Reminder, food parameters were collected retrospectively) resulted in the inability to calculate the consumption amount for all 45 parameters. However, the following 25 parameters were available to calculate the DII:

Energy (kcal), protein (g), total fat (g), vitamin B12 (μg), vitamin B6 (μg), niacin (mg), thiamin (mg), riboflavin (mg), folic acid (μg), vitamin C (mg), vitamin D (μg), fiber (g), caffeine (g), cholesterol (mg), magnesium (mg), vitamin A (retinol equivalent), vitamin E (mg), zinc (mg), selenium (μg), mono-unsaturated fatty acids (MUFA), polyunsaturated fatty acids (PUFA), iron (mg), beta-carotene (μg), carbohydrates (g), and saturated fats (g).

### Calculation and analysis of the dietary inflammatory index (DII)

The original DII is calculated based on the increase, decrease, or no change in six inflammatory markers (C-reactive protein, interleukins 1β, 4, 6, and 10, TNF-α), which are assigned scores of 1+, 1-, or 0, respectively. To calculate the DII, the global average of each dietary parameter was subtracted from the intake level of each dietary parameter in the study population, and the result was divided by its global standard deviation, and a Z-score was computed. This value was then transformed into a percentile-based score, which was multiplied by 2 and subtracted by 1. After obtaining the consumption amount of each dietary parameter for the individual under investigation from the N^−4^ software output, to obtain the DII score for an individual’s dietary pattern, the percentile-based score of each dietary parameter was multiplied by the “overall inflammatory effect score” of that dietary parameter to obtain the “dietary parameter-specific DII score” for the person. Finally, the individual-specific scores of all consumed dietary parameters were summed to calculate the “overall DII score” for the individual. It is worth noting that nutritional parameters with a positive overall inflammatory effect score are considered pro-inflammatory factors, contributing to an increase in DII score (pro-inflammatory diet) (39). In contrast, parameters with a negative overall inflammatory effect score are considered anti-inflammatory factors, contributing to a decrease in the DII score (anti-inflammatory diet). For DII analysis, the continuous DII score and quartiles of the final score for each individual can be utilized (16). In our study, the DII score was calculated based on the consumption of 100 units of each dietary parameter, and the analysis was performed using the continuous score.

### Covariates measurement

Other measured ascertained covariates included: gender, level of education, marital status, smoking status, the socio-economic self-expression status of childhood, socio-economic self-expression status, the status of self-expression of socio-economic fluctuation during life, asset, social activity (using categorical principal components analysis (CAT PCA), the components of social activities, including using the Internet, going to concerts, movies, theaters and restaurants, air travel, reading non-curricular and non-professional books, domestic and foreign non-pilgrimage trips; were combined), and underlying diseases (including Diabetes, Liver problems, Hypothyroidism, and cardiovascular disease (CVD)). These variables were controlled as potential confounders.

### Statistical analysis

Generalized linear models typically use the maximum likelihood or restricted maximum likelihood method for estimating parameters. However, the Generalized Linear Mixed Models (GLMMs) involve more complex random-effects variance components, so there is no closed form for the log-likelihood, making the estimation intractable. Additionally, conventional fitting methods, such as maximum likelihood, may suffer from estimate inflation (sparse-data bias) or fail to converge. Several methods were available as solutions to approximate the likelihood. These methods include the Laplace approximation (LA), the penalized quasi-likelihood (PQL), and the adaptive Gauss-Hermite quadrature (AGHQ) (40–42).

Given the nested association between variables, the large number of nutrients (25 nutrients) and foods (119 foods) in this study, and the inclusion of individual, social, and clinical factors (13 factors) in the models, multilevel linear regression models using GLIMMIX and the Penalized Quasi-Likelihood estimation method were employed to analyze this study. The Empirical-Bayes method, which is the default in the GLIMMIX analysis, was used to estimate a common variance ( $\tau^{2}$ ) for the random coefficients based on the data (33).

The present study utilized a three-level GLIMMIX model, where the first, second, and third levels corresponded to food items, nutrients, and DII, respectively. This is because the effect of foods (level one) depends on the presence of nutrients (level two), and the impact of nutrients depends on their anti-inflammatory properties (level three). The multilevel model used in this study differs from the commonly employed multilevel methods in statistical analysis. Because the model used in this study involves nested independent variables (DII, nutrients, and foods), it differs from studies in which the units of analysis (such as patients, doctors, hospitals) are hierarchical. On the other hand, second-level information is collected based on first-level variables, while third-level information is collected based on second-level variables. While in the usual multilevel analysis, the information from all levels is measured at the individual level.

Independent sample t-test, one-way ANOVA, and simple linear regression models were used to investigate the association between mental disorders and covariates. Conventional analysis was performed using stata-17 software, and GLIMMIX analysis was conducted using SAS-13 software.

### Financial support and sponsorship

This study has been ethically approved by the Ethics Committee of Tehran University of Medical Sciences with the following code numbers: IR.TUMS.MEDICINE.REC.1400.892.

## Results

### Baseline characteristics

A total of 3,501 individuals participated in this study, with 1,362 (38.9%) being men and 2,139 (61.1%) being women. The mean scores of stress, anxiety, and depression were significantly higher in women. Regarding underlying diseases, 5.5% of the individuals had liver diseases, 11.5% had hypothyroidism, 6.9% had cardiovascular disease (CVD), and 3.4% had diabetes. The status of mental disorders was such that the mean scores of stress, anxiety, and depression were higher in individuals with the mentioned diseases than those without the diseases. Regarding smoking status, the results showed that individuals who neither currently smoked nor were exposed to secondhand smoke had the lowest stress and anxiety scores. Conversely, individuals who currently smoked and were exposed to secondhand smoke had the highest stress and anxiety scores (Table 1).

**Table 1:** Baseline characteristics of the participants and their association with mental disorders.

Variable	Frequency number (%)	Stress		Anxiety		Depression	
		Mean ± SD	*P*-value	Mean ± SD	*P*-value	Mean ± SD	*P*-value
Gender^a^							
Male	1,362 (38.9%)	12.72 ± 8.73	<0.001	5.85 ± 5.77	0.004	7.44 ± 7.76	<0.001
Female	2,139 (61.1%)	14.10 ± 9.15		6.43 ± 5.86		8.43 ± 8.20	
Level of education^b^							
Illiterate	18 (0.5%)	9.06 ± 6.80	<0.001	6.50 ± 5.63	<0.001	8.50 ± 6.53	<0.001
Elementary school	129 (3.7%)	13.12 ± 8.96		7.08 ± 5.37		9.01 ± 8.12	
Middle school	152 (4.3%)	13.66 ± 9.64		7.16 ± 6.66		9.28 ± 8.96	
High school diploma	656 (18.7%)	14.63 ± 9.67		7.32 ± 6.39		8.96 ± 8.79	
Associate degree	290 (8.3%)	14.11 ± 9.04		6.69 ± 6.33		8.30 ± 8.30	
Bachelor’s degree	1,338 (38.2%)	13.73 ± 8.90		5.98 ± 5.57		8.19 ± 8.00	
Master’s degree	731 (20.9%)	12.84 ± 8.61		5.61 ± 5.68		7.10 ± 7.34	
PhD and above	187 (5.3%)	11.40 ± 7.98		4.08 ± 3.66		5.30 ± 6.07	
Marital status^b^							
Single (never married)	562 (16.1%)	13.57 ± 9.37	0.349	6.07 ± 6.18	0.170	9.33 ± 9.05	<0.001
Married	2,845 (81.3%)	13.53 ± 8.94		6.19 ± 5.77		7.72 ± 7.74	
Divorced	67 (1.9%)	15.54 ± 8.92		7.19 ± 5.40		10.21 ± 9.48	
Widowed	27 (0.8%)	13.19 ± 9.04		8.07 ± 5.81		9.48 ± 9.90	
Smoking status^b^							
Nonsmokers, non-secondhand smokers	2,377 (67.9%)	12.98 ± 8.86	<0.001	5.69 ± 5.47	<0.001	7.42 ± 6.01	<0.001
Exclusive cigarette smokers, non-secondhand smokers	50 (1.4%)	13.88 ± 10.03		5.92 ± 5.24		7.36 ± 7.32	
Exclusive water pipe smokers, non-secondhand smokers	183 (5.2%)	15.21 ± 8.54		7.66 ± 6.21		9.78 ± 8.39	
Concurrent smokers, non-secondhand smokers	24 (0.7%)	15.50 ± 11.18		9.42 ± 8.87		11.25 ± 11.74	
Non-smokers, secondhand smokers	433 (12.4%)	14.74 ± 8.93		7.02 ± 5.96		8.98 ± 8.26	
Exclusive cigarette smokers, secondhand smokers	264 (7.5%)	14.01 ± 9.24		6.98 ± 6.59		8.89 ± 8.51	
Exclusive water pipe smokers, secondhand smokers	96 (2.7%)	15.71 ± 10.21		7.41 ± 6.01		11.06 ± 9.95	
Concurrent smokers, secondhand smokers	74 (2.1%)	16.59 ± 9.57		9.24 ± 8.27		10.64 ± 9.22	
The childhood socio-economic status^b^							
Above	149 (4.3%)	13.98 ± 8.51	0.056	6.93 ± 6.61	<0.001	8.99 ± 9.20	<0.001
Medium upward	607 (17.3%)	13.10 ± 8.90		5.62 ± 5.59		7.02 ± 7.25	
Medium	1719 (49.1%)	13.33 ± 8.93		6.13 ± 5.82		7.79 ± 7.86	
Medium downward	556 (15.9%)	13.94 ± 9.09		6.12 ± 5.64		8.31 ± 8.03	
Down	470 (13.4%)	14.51 ± 9.48		7.12 ± 6.03		9.66 ± 9.00	
The socio-economic self-expression status^b^							
Above	156 (4.5%)	10.78 ± 7.89	<0.001	4.64 ± 5.64	<0.001	5.06 ± 7.29	<0.001
Medium upward	924 (26.4%)	13.28 ± 9.15		5.85 ± 5.83		7.10 ± 7.15	
Medium	1878 (53.6%)	13.49 ± 8.86		6.15 ± 5.73		8.03 ± 7.98	
Medium downward	417 (11.9%)	14.64 ± 9.11		7.04 ± 5.81		9.69 ± 8.05	
Down	126 (3.6%)	16.94 ± 9.98		8.79 ± 6.49		13.28 ± 10.19	
Fluctuations in socioeconomic status throughout one’s life^b^							
A lot	147 (4.2%)	16.63 ± 9.53	<0.001	8.44 ± 5.93	<0.001	11.74 ± 8.32	<0.001
Much	469 (13.4%)	15.77 ± 9.25		7.38 ± 6.05		9.61 ± 8.67	
Neither too much nor too little	786 (22.5%)	13.71 ± 8.89		6.45 ± 6.25		8.05 ± 8.04	
Little	662 (18.9%)	13.31 ± 8.83		5.87 ± 5.56		7.87 ± 7.77	
very little	1,437 (41%)	12.59 ± 8.85		5.61 ± 5.50		7.22 ± 7.76	
Asset^c^ (Regression coefficient ± SE)		−0.27 ± 0.14	0.069	−0.33 ± 0.09	0.001	−0.28 ± 0.13	0.031
Social Activity^c^ (Regression coefficient ± SE)		−0.51 ± 0.15	0.001	−0.31 ± 0.09	0.002	−0.57 ± 0.13	<0.001
Liver problems^a^							
Yes	185 (5.3%)	15.03 ± 9.47	0.024	7.21 ± 5.79	0.016	9.46 ± 8.66	0.014
No	3,316 (94.7%)	13.49 ± 8.98		6.15 ± 0.83		7.96 ± 8.00	
Hypothyroidism^a^							
Yes	401 (11.5%)	14.67 ± 9.39	0.009	6.77 ± 6.10	0.041	8.65 ± 8.35	0.108
No	3,100 (88.5%)	13.43 ± 8.96		6.13 ± 5.79		7.96 ± 8.00	
CVD ^a^							
Yes	243 (6.9%)	14.76 ± 9.24	0.033	7.30 ± 5.82	0.002	8.55 ± 8.36	0.309
No	3,258 (93.1%)	13.48 ± 8.99		6.12 ± 5.82		8.00 ± 8.02	
Diabetes^a^							
Yes	150 (4.3%)	14.33 ± 9.77	0.295	7.35 ± 5.95	0.014	8.13 ± 7.53	0.885
No	3,351 (95.7%)	13.54 ± 8.98		6.15 ± 5.82		8.04 ± 8.07	

^a^Independent sample *T* test.

^b^One-way ANOVA.

^c^Simple linear regression model.

### Conventional analysis

The mean scores of stress, anxiety, and depression were 13.57 ± 9.01 (95% CI = 13.27, 13.87), 6.20 ± 5.83 (95% CI = 6.01, 6.39), and 8.04 ± 8.04 (95% CI = 7.77, 8.30), respectively. The mean DII score was-0.01 ± 0.43 (95% CI = −0.02, 0.00), with a minimum of-1.06 and a maximum of 5.37.

Table 2 demonstrates that the simple conventional models, without adjustment for potential confounding variables, for each unit increase in the DII score, the mean scores of stress, anxiety, and depression increased by 3.439 (95% CI = 2.759, 4.119; *p* < 0.001), 2.814 (95% CI = 2.378, 3.250, p < 0.001), and 2.562 (95% CI = 1.952, 3.171, p < 0.001), respectively. It also shows that in the multiple conventional model, with adjustment for baseline characteristics, nutrients, and foods, for each unit increase in the DII score, the mean scores of stress, anxiety, and depression increased by 11.521 (95% CI = 2.320, 20.723, *p* = 0.014), 6.389 (95% CI = 0.594, 12.184, *p* = 0.031), and 5.240 (95% CI = −2.909, 13.390, *p* = 0.207), respectively (Table 2).

**Table 2:** Association between DII and mental disorders adjusted by baseline characteristics, nutrients and foods and the comparison between conventional and GLIMMIX models.

Models	Exposure	Stress				Anxiety				Depression			
		Coe.^a^	95% Confidence interval		P-value	Coe.^a^	95% Confidence interval		*P*-value	Coe.^a^	95% Confidence interval		*P*-value
			Lower	Upper			Lower	Upper			Lower	Upper	
Simple conventional model^b^	DII	3.439	2.759	4.119	<0.001	2.814	2.378	3.250	<0.001	2.562	1.952	3.171	<0.001
Multiple conventional model^c^	DII	11.521	2.320	20.723	0.014	6.389	0.594	12.184	0.031	5.240	−2.909	13.390	0.207
GLIMMIX^d^	DII	3.552	3.170	4.533	<0.001	4.264	0.066	8.462	0.046	3.021	2.418	3.623	<0.001

^a^Regression coefficient.

^b^Simple linear regression models without adjustment for potential confounders.

^c^Multiple linear regression models with adjustment for baseline characteristics, nutrients and foods.

^d^Generalized linear mixed models.

### Multilevel analysis via GLIMMIX

Based on the model, which its results are shown in Table 2, after adjusting for baseline characteristics, nutrients, and foods, DII still has an association with mental disorders. For each unit increase in the DII score, the mean scores of stress, anxiety, and depression increased by 3.552 (95%CI = 3.170, 4.533; *p*<0.001), 4.264 (95%CI = 0.066, 8.462; *p* = 0.046), and 3.021 (95%CI = 2.418, 3.623; *p*<0.001), respectively (Table 2).

In multilevel model, having liver problems, being female and the status of socio-economic fluctuation during life increase the mean scores of mental disorders, while increasing social activity and socio-economic level decrease the mean scores of mental disorders (Table 3).

**Table 3:** Relationship between baseline characteristics of the participants and mental disorders with GLIMMIX modela.

Variables	measure	Stress				Anxiety				Depression			
		Coe.^b^	95% Confidence Interval		*P*-value	Coe.^b^	95% Confidence Interval		*P*-value	Coe.^b^	95% Confidence Interval		*P*-value
			Lower	Upper			Lower	Upper			Lower	Upper	
Liver problems	Yes/NO	1.878	0.573	3.182	0.004	1.289	0.481	2.097	0.001	1.935	0.781	3.088	0.001
Hypothyroidism	Yes/NO	0.778	−0.154	1.711	0.102	0.404	−0.172	0.982	0.169	0.406	−0.418	1.230	0.334
CVD	Yes/NO	1.176	0.033	2.319	0.043	1.216	0.509	1.923	<0.001	0.572	−0.437	1.583	0.266
Diabetes	Yes/NO	0.367	−1.069	1.803	0.616	1.219	0.324	2.114	0.007	−0.119	−0.437	1.583	0.853
Gender	Female/ Male	2.735	2.073	3.397	<0.001	2.145	1.690	2.600	<0.001	2.471	1.887	3.056	<0.001
Level of education	Per one degree	−0.078	−0.312	0.154	0.508	−0.337	−0.487	−0.188	<0.001	−0.345	−0.551	−0.139	0.001
Marital status	Per change of each level (According to Table 1)	0.059	−0.619	0.737	0.864	0.239	−0.183	0.662	0.266	−0.952	−1.551	−0.353	0.001
Smoking status	Per change of each level (According to Table 1)	0.349	0.031	0.666	0.031	0.160	−0.036	0.357	0.109	0.373	0.092	0.653	0.009
The childhood socio-economic status	Per decrease of each level (According to Table 1)	0.900	0.500	1.300	<0.001	0.463	0.215	0.711	<0.001	1.413	1.059	1.766	<0.001
The socio-economic self-expression status	Per decrease of each level (According to Table 1)	−0.740	−0.981	−0.498	<0.001	−0.364	−0.514	−0.214	<0.001	−0.547	−0.761	−0.333	<0.001
Fluctuations in socioeconomic status throughout one’s life	Per decrease of each level (According to Table 1)	0.432	0.286	0.579	<0.001	0.267	0.175	0.359	<0.001	0.408	0.278	0.538	<0.001
Asset	Per one score	−0.064	−0.377	0.249	0.686	0.003	−0.197	0.201	0.973	0.220	−0.056	0.497	0.118
Social activity	Per one score	−0.389	−0.695	−0.082	0.012	−0.074	−0.266	0.117	0.445	−0.278	−0.549	0.007	0.043

^a^Adjusted for nutrients, foods, and DII.

^b^Regression coefficient.

## Discussion

In this study, 3,501 (98.61%) out of 3,550 participants whose information was completed in phase 1 of the TEC study and who met the eligibility criteria were included in the study. The study aimed to investigate the relationship between the DII and mental disorders using a multilevel, three-level model with the GLIMMIX macro in the SAS software. The results revealed that an increased DII score is associated with higher levels of stress, anxiety, and depression. Considering the difference in regression coefficients between conventional and multilevel models, it can be concluded that multilevel models provide more accurate and valid estimates.

In our study, the results of conventional models showed that after adjusting for potential confounders, an increase of one unit in the DII score was associated with an increase in the average scores of stress, anxiety, and depression. The increases were 11.521 (with a 95% confidence, this value can vary between 2.320 and 20.723, *p* = 0.014), 6.389 (with a 95% confidence, this value can vary between 0.594 and 12.184, *p* = 0.031), and 5.240 (with a 95% confidence, this value can vary between-2.909 and 13.390, *p* = 0.207), respectively. These results are consistent with various studies (26, 43–46). However, previous studies and conventional models have shown that the resulting confidence interval is wide, indicating low accuracy and plausibility. Diets can affect six inflammatory biomarkers in the body: interleukin 1β, interleukin 4, interleukin 6, interleukin 10, TNF-α, and C-reactive protein (CRP). A pro-inflammatory diet can significantly increase the levels of interleukin 1β, interleukin 6, TNF-α, or CRP biomarkers, while decreasing the levels of interleukin 4 or interleukin 10 biomarkers. The anti-inflammatory diet can also significantly decrease the biomarkers of interleukin 1β, interleukin 6, TNF-α, and CRP, or increase the biomarkers of interleukin 4 and interleukin 10 (16). The hyperactivity of the hypothalamic–pituitary–adrenal (HPA) axis and dysregulation of the immune system are the underlying causes of irregularities in the activity of the kynurenine pathway. Its basic role in a healthy organism is to transform tryptophan into two essential compounds engaged in mood regulation: serotonin and melatonin. Based on the kynurenine pathway hypothesis of depression etiology, inflammatory factors cause excessive activation of indoleamine-2, 3-dioxygenase (IDO), an enzyme present in microglia, astrocytes, and neurons. This enzyme catabolizes tryptophan, the precursor of serotonin, into kynurenine (KYN), a neurotoxic metabolite that increases the risk of neurodegenerative and neurotoxic processes. In this way, IDO reduces the amount of tryptophan available for the production of serotonin, which is directly linked to the etiology of depression (47–49).

The results of multilevel models showed that an increase of one unit in DII is associated with an increase in the average scores of stress, anxiety, and depression. The increases were 3.552 (With a 95% confidence, this value can vary between 3.170 and 4.533, p<0.001), 4.264 (With a 95% confidence, this value can vary between 0.066 and 8.462, p<0.001), and 3.021 (with a 95% confidence, this value can vary between 2.418 and 3.623, p<0.001) points, respectively. Comparing the results of conventional and multilevel models, we found that the standard error and confidence interval of coefficients in multilevel models were lower. This indicates that the effects obtained from the multilevel model were more accurate and plausible than those obtained from conventional models. According to the upper and lower limits of the 95% confidence intervals, and without relying solely on the *p*-value, it can be concluded that the association between DII and mental disorders is clinically important (50–52).

The main components of socio-economic status (job, income, and education) have a positive and significant relationship with mental disorders such as depression, stress, and anxiety (53); in terms of the association between socio-economic status and mental disorders, the results of our study showed that individuals with a low childhood socioeconomic status had the highest average scores of mental disorders, while those with a medium-high childhood socioeconomic status had the lowest average scores. Similarly, individuals with a currently low social level and high economic status had the highest and lowest average scores of mental disorders, respectively. Furthermore, the association between fluctuations in socioeconomic status throughout one’s life and the occurrence of mental disorders revealed that individuals who experienced the most fluctuations had the highest average score of mental disorders, while those who experienced the least fluctuations had the lowest average score of mental disorders; this finding is consistent with previous studies that have demonstrated a correlation between childhood socioeconomic status and the occurrence, duration, and intensity of mental disorders (54).

Since anti-inflammatory and pro-inflammatory foods are consumed together, studying the relationship between DII and mental health using conventional analysis methods is not feasible (17). Conventional analyses cannot provide accurate estimates of the effects of different foods with similar levels of nutrients in epidemiological studies. This bias in estimating effects occurs due to small-sample bias or instability in maximum-likelihood estimates (33).

Based on the conducted studies and the possible consumption levels of each parameter at the global level, the maximum DII score is +7.98 (corresponding to the maximum consumption of pro-inflammatory parameters), and the minimum DII score is-8.87 (corresponding to the minimum consumption of pro-inflammatory parameters and the maximum consumption of anti-inflammatory parameters) (16). In our study, the maximum and minimum DII scores were 5.37 and-1.06, respectively, which can be attributed to the differences in dietary style and the number of parameters used in calculating DII. DII consists of 45 food parameters, but we used 25 parameters in our study to calculate DII. One of the characteristics of DII is its flexibility in increasing or decreasing the number of components in different studies. However, if the necessary data is available, using the complete version of DII can provide a better and more comprehensive understanding of its relationship with mental disorders.

From the perspective of the importance of using a multilevel model in this study, it should be noted that Generalized Linear Models (GLMs) are employed when the data lacks independence. When data are collected from different levels or clusters (e.g., food clusters, nutrients), there is an intra-cluster correlation between units of the same level and a correlation between units of different levels. Consequently, due to correlated data, using GLMs is inappropriate since they assume that observations within the study units are independent. If a GLM is applied to nested data, the independence assumption is seriously violated, leading to an underestimation of standard errors and the identification of significant spurious relationships (55).

When correlation exists between the data, instead of GLMs, GLMMs are utilized. GLMMs, in fact, a statistical model, are an extension of GLMs where random effects with a normal distribution are added. GLMMs incorporate random effects into the linear predictor or directly model the existing correlation in the data (56).

Multilevel modeling can provide more accurate and plausible effects estimates than conventional models (57). The results of the current study also demonstrated that the effects obtained from the multilevel model were more accurate and plausible than those obtained from conventional models.

Numerous studies have shown that multilevel models can perform statistically better than conventional approaches in the analysis of epidemiological data in the face of multiple exposures (58–72). This improved performance is partly because higher levels of a multilevel model incorporate additional information for estimation. For example, in a practical epidemiological study of nutrition, a multilevel model improved upon conventional estimates by shrinking the effects of food items on each other when those foods had similar levels of nutrients (62).

Most studies and published articles on applying multilevel models have been conducted using two-level models. However, these models can be extended to include more levels based on the study’s objectives (32). In the present study, considering the available data and the main purpose of examining the relationship between DII and mental disorders, a three-level multilevel model was employed, including foods, nutrients, and DII levels. Two-level models include a residual effect for the first level (33). Still, in the three-level model used in our study, two residual effects [one for the first level (δ_j_) and one for the second level (θ_i_)] are incorporated into the model.

The strengths of this study include the use of multilevel models with three levels, which allowed for the adjustment of the effects of foods and nutrients. This adjustment helped to obtain more accurate and reliable estimates while reducing potential confounding biases and collinearity effects. The lack of necessary nutritional data to calculate the DII with more parameters was one of the limitations of this study.

## Conclusion

Considering the nature of the data and the nested relationship between DII, nutrients, and foods, we needed to use a three-level multilevel model. Due to the differences in regression coefficients between conventional and multilevel models, epidemiologists, particularly in nutritional studies with nested data, must employ multilevel models to obtain more precise and credible estimates. Considering that increasing the DII score is associated with higher scores of mental disorders such as depression, stress, and anxiety, it is recommended to minimize the consumption of pro-inflammatory nutrients and foods. This can help prevent and reduce the possibility of developing mental disorders. The fluctuations in socioeconomic status throughout one’s life, socio-economic self-expression, and social activity have a significant relationship with the mean scores of mental disorders. Additionally, suffering from underlying diseases such as diabetes, liver problems, and cardiovascular disease can disrupt mental health.

## Data availability statement

The original contributions presented in the study are included in the article/supplementary material, further inquiries can be directed to the corresponding author.

## Ethics statement

The studies involving humans were approved by the Ethics Committee of Tehran University of Medical Sciences. The studies were conducted in accordance with the local legislation and institutional requirements. The participants provided their written informed consent to participate in this study.

## Author contributions

RB: Writing – original draft, Data curation, Formal analysis, Software, Writing – review & editing. MM: Writing – review & editing, Investigation, Methodology, Validation. FV: Writing – review & editing, Investigation, Validation. A-AN: Writing – review & editing, Investigation, Validation. SN: Writing – review & editing, Investigation, Methodology, Project administration, Validation.
